# Structure-Guided Creation of an Anti-HA Stalk Antibody F11 Derivative That Neutralizes Both F11-Sensitive and -Resistant Influenza A(H1N1)pdm09 Viruses

**DOI:** 10.3390/v13091733

**Published:** 2021-08-31

**Authors:** Osamu Kotani, Yasushi Suzuki, Shinji Saito, Akira Ainai, Akira Ueno, Takuya Hemmi, Kaori Sano, Koshiro Tabata, Masaru Yokoyama, Tadaki Suzuki, Hideki Hasegawa, Hironori Sato

**Affiliations:** 1 Center for Pathogen Genomics, National Institute of Infectious Diseases, Tokyo 208-0011, Japan; yokoyama@nih.go.jp (M.Y.); hirosato@nih.go.jp (H.S.); 2 Center for Influenza and Respiratory Virus Research, National Institute of Infectious Diseases, Tokyo 208-0011, Japan; yasuzuki@nih.go.jp (Y.S.); hasegawa@nih.go.jp (H.H.); 3 Department of Pathology, National Institute of Infectious Diseases, Tokyo 162-8640, Japan; ainai@nih.go.jp (A.A.); a-ueno@nih.go.jp (A.U.); t-hemmi@nih.go.jp (T.H.); ka--sano@nih.go.jp (K.S.); k-tabata@czc.hokudai.ac.jp (K.T.); tksuzuki@nih.go.jp (T.S.); 4 Division of Molecular Pathobiology, International Institute for Zoonosis Control, Hokkaido University, Hokkaido 001-0020, Japan

**Keywords:** influenza virus, anti-HA stalk antibody, molecular interactions, molecular dynamics simulation, antibody modification, neutralization assay

## Abstract

The stalk domain of influenza virus envelope glycoprotein hemagglutinin (HA) constitutes the axis connecting the head and transmembrane domains, and plays pivotal roles in conformational rearrangements of HA for virus infection. Here we characterized molecular interactions between the anti-HA stalk neutralization antibody F11 and influenza A(H1N1)pdm09 HA to understand the structural basis of the actions and modifications of this antibody. In silico structural analyses using a model of the trimeric HA ectodomain indicated that the F11 Fab fragment has physicochemical properties, allowing it to crosslink two HA monomers by binding to a region near the proteolytic cleavage site of the stalk domain. Interestingly, the F11 binding allosterically caused a marked suppression of the structural dynamics of the HA cleavage loop and flanking regions. Structure-guided mutagenesis of the F11 antibody revealed a critical residue in the F11 light chain for the F11-mediated neutralization. Finally, the mutagenesis led to identification of a unique F11 derivative that can neutralize both F11-sensitive and F11-resistant A(H1N1)pdm09 viruses. These results raise the possibility that F11 sterically and physically disturbs proteolytic cleavage of HA for the ordered conformational rearrangements and suggest that in silico guiding experiments can be useful to create anti-HA stalk antibodies with new phenotypes.

## 1. Introduction

Infection with the influenza virus often causes serious respiratory diseases in humans, and thus presents a persistent public health threat. Although vaccination is a promising strategy to suppress the magnitude of an influenza epidemic, current vaccines rarely overcome the genetic diversity of influenza viruses. The major immunodominant region for raising neutralization antibodies is located in a highly variable head domain of envelope glycoprotein hemagglutinin (HA) [1,2,3,4]. Consequently, neutralization antibodies raised against the head domain are usually strain-specific, and often drive the selection of new variants that are less sensitive to the circulating antibodies [5]. Therefore, antibodies that target more conserved regions have been sought to combat infection by influenza viruses [6,7,8].

Monoclonal antibodies that target the stalk domain of the HA protein have been shown to protect mice against a broad range of influenza viruses [9]. Moreover, viruses that acquired resistance to the anti-HA stalk antibodies were attenuated in mice and could be controlled with a candidate vaccine [10]. The stalk domain is located in an extracellular portion of the HA protein, and is a key structural unit for the pH-dependent conformational changes of the HA protein to gain membrane fusion activity for genome uncoating in the endosome, thereby playing essential roles in establishing virus infection in the cells [11]. The stalk domain is relatively more conserved within and among influenza virus strains in nature, as compared with the HA globular region [2,4]. This may imply that the stalk domain is under strong functional/structural constraints against changes. Thus, antibodies targeting the stalk domain can be beneficial tools to combat infections of highly mutable influenza viruses [6,7,8]. To date, many anti-HA stalk antibodies have been isolated and characterized for their binding modes to HA protein [9,12,13,14,15,16,17]. Interestingly, about half of these antibodies bind to HA in a manner that crosslinks two HA monomers of the HA trimer on the virion [9,12,13,14]. Based on the structural information, the anti-HA stalk antibodies have been proposed to inhibit conformational changes of the HA protein that establish the infections of target cells [9,12,13,14,15,16,17].

Recently, we independently isolated an anti-HA stalk antibody, termed F11, from an individual inoculated with an intranasal inactivated influenza vaccine [18]. In its IgA form, F11 was able to neutralize the H1N1, H3N2, and H5N1 strains of influenza A, but in its IgG form it had lower cross-reactivity [18]. We also isolated variants of influenza A(H1N1)pdm09 that exhibited reduced susceptibilities to neutralization mediated by the F11 antibody [18]. We found that single substitutions near the hydrophobic cleft of the HA stalk region could confer F11 resistance to the virus in association with changes in the physicochemical properties of the hydrophobic cleft [18]. However, the binding mode of F11 to HA and therefore the molecular mechanisms underlying the F11-mediated neutralization remain unknown. To address each of these issues, we herein examined the structural basis of molecular interactions between the F11 Fab fragment and glycosylated HA trimer using in silico techniques involving molecular dynamics (MD) simulations. We then used the obtained information to create a F11 variant that can neutralize both F11-sensitive and F11-resistant influenza A(H1N1)pdm09 viruses.

## 2. Materials and Methods

### 2.1. Molecular Modeling of the Trimeric HA Ectodomain of Influenza A(H1N1)pdm09

A three-dimensional model of the HA trimer ectodomain of the A/Narita/1/2009 (H1N1)pdm09 virus in the ligand-free state was first constructed by the homology modeling method using the reported amino acid sequence of HA of A/Narita/1/2009 (H1N1)pdm09 [19] (Figure S1). The Molecular Operating Environment (MOE) (Chemical Computing Group Inc., Montreal, QC, Canada) was used for the homology modeling. A high resolution crystal structure of an HA trimer of the A/California/04/2009 (H1N1) virus (PDB code: 3LZG, a resolution of 2.6 Å) [20] was used as the modeling template. The template represents a structure of the extracellular portion of the H1N1 HA strain. The high-mannose oligosaccharide Man_5_GlcNAc_2_ was attached to potential *N*-glycosylation sites in HA using tools in GLYCAM-Web [21]. Subsequently, the glycosylated HA trimer model was subjected to MD simulation as described for HA trimers of influenza A virus [18,22]. Briefly, the simulations were performed using the pmemd.cuda.MPI module in the Amber 16 program package [23] with the *ff14SB* force field for simulation of protein [24], with the *GLYCAM06j-1* force field for simulation of glycan [25], and with the TIP3P water model for simulation of aqueous solutions [26]. A non-bonded cut-off of 10 Å was used. Bond lengths involving hydrogen were constrained with SHAKE, a constraint algorithm that satisfies Newtonian motion [27]. The time step for all MD simulations was set to 2 fs. After heating calculations performed for 20 ps up to 310 K using the NVT ensemble, simulations were executed for 400 ns in 150 mM NaCl using the NPT ensemble (at 1 atm, 310 K).

### 2.2. Docking Simulations of the Glycosylated HA Ectodomain and Anti-HA Stalk Antibody F11

A three-dimensional model of the stalk antibody F11 Fab fragment was constructed using the Antibody Modeler application of MOE [28]. Amino acid sequences of the antibody F11 immunoglobulin light chain (GenBank accession number: QII15722) and heavy chain (GenBank accession number: QII15721) were used for modeling of the Fab fragment (Figure S1A) [18]. The obtained models were optimized by energy minimization using the Amber10: Extended Huckel Theory (EHT) force field implemented in MOE. Docking simulations were performed using the Dock application of MOE [29,30]. Briefly, amino acid residues in the HA stalk domain (HA1: 18–57; HA2: 345–515) (Figure S1B) from a structure of the glycosylated HA trimer at 400 ns of MD simulation were defined as a receptor for binding of stalk-antibody F11, while an energy minimized structure of a variable region of the F11 Fab fragment was defined as a ligand for binding to the HA stalk domain of the glycosylated-HA trimer. Subsequently, physicochemically possible docking poses between the receptor and ligand moieties were comprehensively searched with the Dock application. The docking simulations were executed to collect 100 possible docking poses. Binding energies of the F11 Fab fragment to the glycosylated-HA trimer ectodomain were calculated with the Dock tool using individual docking poses.

### 2.3. MD Simulation of the Glycosylated HA Trimer Docked to the F11 Fab Fragment

The complex model obtained by in silico docking simulation was subjected to MD simulation under the conditions described previously with HA trimers of influenza A virus [18,22], which were essentially the same as the conditions used for MD simulations of HIV-1 structural proteins such as capsid [31] and envelope gp120 bound to CD4 and CCR5 [32]. Briefly, MD simulations were performed using the pmemd.cuda.MPI module in the Amber 16 program package [23] with the *ff14SB* force field for simulation of protein [24], with the *GLYCAM06j-1* force field for simulation of glycan [25] and with the TIP3P water model for simulation of aqueous solutions [26]. A non-bonded cut-off of 10 Å was used. Bond lengths involving hydrogen were constrained with SHAKE, a constraint algorithm that satisfies Newtonian motion [27]. The time step for all MD simulations was set to 2 fs. After heating calculations performed for 20 ps up to 310 K using the NVT ensemble, simulations were executed for 200 ns in 150 mM NaCl using the NPT ensemble (at 1 atm, 310 K).

### 2.4. Calculation of Root Mean Square Deviation (RMSD)

Calculations of RMSDs were done as described previously [32,33]. Briefly, RMSDs between the heavy atoms of the initial complex structure and the structures at given time points of the MD simulation were calculated to monitor the overall structural changes during the MD simulations using the cpptraj module in AmberTools 16 [23].

### 2.5. Calculation of Root Mean Square Fluctuation (RMSF)

The trajectory files during last 20 ns of MD simulations (*n* = 10,000) were used to calculate RMSF. RMSF of the Cα atoms of amino acid residues was calculated to obtain information regarding the atomic fluctuations of individual amino acid residues of HA protein during MD simulations as described previously [18,31,32,33] using the tools in Amber 16 [23]. Briefly, the average structures during the last 20 ns of MD simulations were used as reference structures for RMSF calculation. RMSF, which quantifies differences between average values and those obtained at a given time point of MD simulation, was calculated using the ptraj module, a trajectory analysis tool in AmberTools 16 [23].

### 2.6. Analysis of Noncovalent Interaction Sites and Networks between HA and F11

Analyses of noncovalent intermolecular interactions for F11 antibody binding to the HA protein during MD simulations were performed as described previously [33]. Briefly, noncovalent interactions between the F11 antibody and HA protein were identified using the Contact Analysis application of MOE. This application allows identification of distance-dependent noncovalent interactions between atoms, such as hydrogen bonds, Van der Waals interactions, π-interactions, and ionic interactions. HA-F11 complex structures (*n* = 20) collected at every 1 ns during 180 to 200 ns after MD simulations were used for the search of interaction sites. Noncovalent interaction networks in the HA/F11 complex detected in the 20 complex structures were visualized using Cytoscape software version 3.8.2 [34], a program for integrated models of biomolecular interaction networks.

### 2.7. Protein Patch Analysis

Assessment and visualization of hydrophobic patches on the HA-F11 complex model were done as described previously [31] using the Protein Patch Analyzer tool in MOE [35,36,37,38]. Briefly, protein hydrophobic patches with a minimum area of 50 Å^2^ for protein-protein interactions [39,40] were identified with the HA-F11 complex structure at 200 ns of MD simulation using the Protein Patch Analyzer.

### 2.8. Shannon Entropy Analysis

Amino acid variation at contact sites of the HA protein was analyzed with Shannon entropy as described previously [31,41]. Amino acid sequences of the HA protein of the influenza A virus H1 (*n* = 36,564; former seasonal H1N1 and H1N1pdm) and H3 (*n* = 53,989; H3N2 and H3N2v) were obtained from the GISAID database [42]. Shannon entropy was calculated on the basis of Shannon’s equation [43]:

$$H\left(i\right)=-\underset{x_{i}}{\sum }p\left(x_{i}\right)\log_{2}p\left(x_{i}\right)\;\;\;\;\;\;\;\;\;\;\;\;\;\;\;\;\;\;\;\;\;\;\left(x_{i}=G,A,I,V,\ldots \right)$$

where *H*(*i*), *p*(*x_i_*), and *i* indicate the amino acid entropy score for an individual position, the probability of occurrence of a given amino acid at that position, and the number of the position, respectively. An *H*(*i*) score of zero indicates absolute conservation, whereas a score of 4.4 bits indicates complete randomness.

### 2.9. In Silico Site-Directed Mutagenesis of the F11 Antibody

F11-HA complex structures obtained during 180 to 200 ns after MD simulations were used for site-directed mutagenesis of the F11 antibody. For the comprehensive mutagenesis study, F11 residues forming noncovalent bonds with HA glycan or amino acid were identified with the structures during 180 to 200 ns of MD simulation using the Contact Analysis application of MOE. Subsequently the contact residues and surrounding residues of F11 were individually substituted with 19 nonself amino acid residues. Modeling of the F11 mutants and calculation of changes in the F11 stability and binding affinity to HA by the mutations were done using the Protein Design application of MOE, along with the MM/GBVI program to calculate the binding affinity as described previously [44,45,46]. The results were expressed by the change in the free energy of binding (ΔΔG). In some cases, binding energies of individual complexes of F11-HA obtained during MD simulations were calculated using the Potential Energy application of MOE to assess the distribution of binding energies in solution.

### 2.10. Cells and Viruses

Madin-Darby canine kidney (MDCK) cells (American Type Culture Collection; CCL-34) were maintained at 37 °C/5% CO_2_ in minimum essential medium (MEM; Life Technologies, Carlsbad, CA, USA) containing 10% fetal bovine serum (Thermo Fisher Scientific, Waltham, MA, USA) and pen-strep mix (100 units/mL penicillin and 100 μg/mL streptomycin; Life Technologies). Humanized MDCK (hCK) cells [47] were maintained at 37 °C/5% CO_2_ in Dulbecco’s Modified Eagle’s Medium-high glucose (DMEM; Sigma-Aldrich, St. Louis, MO, USA) containing 5% fetal bovine serum, pen-strep mix (100 units/mL penicillin and 100 μg/mL streptomycin), 5 µg/mL blasticidin (InvivoGen, San Diego, CA, USA), and 2 µg/mL puromycin dihydrochloride (Life Technologies). Expi293F cells (Thermo Fisher Scientific) were maintained at 37 °C/8% CO_2_ in Erlenmeyer cell culture flasks (Corning, Tewksbury, MA, USA) containing Expi293 Expression Medium (Thermo Fisher Scientific). A/Narita/1/2009 (H1N1)pdm09 virus [19] and A/Narita/1/2009 (H1N1)pdm09-derived F11 escape mutants (C1 and G6 [18]) were propagated in MDCK cells.

### 2.11. Site-Directed Mutagenesis, Expression, and Purification of IgG Antibodies

Plasmids encoding F11 γ1HC and F11 κLC were prepared as previously described [18]. DNA fragments encoding the variable region of the heavy or light chain of each mutant F11 clone were *Homo sapiens* codon-optimized and synthesized by the GenPlus High-throughput Gene Synthesis service (GenScript, Piscataway, NJ, USA). Synthesized DNA fragments were cloned into γ1HC or κLC vectors and used for the site-directed mutagenesis. To generate IgG1, Expi293F cells grown in Expi293 Expression Medium were diluted to 2.5 × 10^6^ cells/mL and transfected with 26 μg of γ1HC and 24 μg of κLC per 50 mL of final culture volume using the ExpiFectamine 293 Transfection Kit (Thermo Fisher Scientific). At 7 days post-transfection, cell culture supernatants were centrifuged at 1200× *g* and filtered to remove cell debris. The supernatants were then purified using CaptureSelect CH1-XL (Thermo Fisher Scientific) according to the manufacturer’s instructions. Purified antibodies were concentrated using Amicon Ultracell (Merck, Darmstadt, Germany) centrifugation units with a cut-off of 30 kDa; the buffer was changed to 20 mM phosphate buffer (pH 7.4) using a Zeba Spin Desalting Column (Thermo Fisher Scientific).

### 2.12. Assessment of Mutated IgG1 Antibody Quality by SDS-PAGE Analysis

To confirm successful production and purification of mutated IgG1 antibodies, purified antibodies were analyzed by SDS-PAGE on 5–20% gradient e-PAGEL gel (ATTO Inc., Tokyo, Japan). Precision Plus Protein Dual Color Standards (Bio-Rad, Hercules, CA, USA) were used as molecular weight markers. The SDS-PAGE gels were stained with Quick-CBB PLUS (FUJIFILM Wako Pure Chemical Corporation, Osaka, Japan) according to the manufacturer’s instructions.

### 2.13. Microneutralization (NT) Assay

The microneutralization assay was performed using hCK cells [47] and 100 TCID_50_ of influenza virus, essentially as previously described [48,49]. Briefly, each sample (serially diluted 2-fold) was mixed with an equal volume of diluent containing influenza virus (equivalent to 100 TCID_50_) and incubated for 30 min at 37 °C. This mixture was added to a monolayer of hCK cells in the wells of a 96-well plate. Four control wells containing virus or diluent alone were included on each plate. The plates were incubated for 5 days at 37 °C/5% CO_2_. All wells were observed for the presence or absence of cytopathic effects under light microscopy, fixed with 10% formalin phosphate buffer for more than 5 min at room temperature, and then stained with Naphthol blue black. After washing and drying, cells were solubilized with 0.1 M NaOH and absorbance (A) was read at 630 nm. The average A630 value was determined from virus-only controls (A virus) and medium only controls (A cell). Values > 50% of the specific signal, calculated using the formula X = (A cell − A virus)/2, were considered positive for neutralization. NT activity was defined as the reciprocal of the lowest concentration (µg/mL) of antibody, at which point A630 was >X.

### 2.14. Statistical Analysis

All statistical analyses were performed using the Prism statistical software package (version 7.0; GraphPad Software). An ordinary one-way ANOVA followed by Dunnett’s multiple comparison test or a Kruskal–Wallis test followed by Dunn’s multiple comparison test was used to analyze each dataset, as indicated in the figure legends. The threshold for statistical significance was set at 5% (*p* < 0.05).

## 3. Results

### 3.1. Molecular Modeling of the Glycosylated HA Trimer Ectodomain Docked to the F11 Fab Fragment

To conduct an in silico structural study of the interactions between the F11 antibody and HA, we constructed a molecular model of the glycosylated HA trimer ectodomain docked to the F11 Fab fragment using homology modeling, molecular dynamics (MD) simulation, and docking simulation as described in Materials and Methods. We first constructed a model of the unliganded HA trimer ectodomain of the A/Narita/1/2009 (H1N1)pdm09 virus [19] used in a previous report [18] and the present study by homology modeling. The high-mannose oligosaccharide Man_5_GlcNAc_2_ was added to the potential *N*-glycosylation sites in the trimeric HA using tools in GLYCAM-Web [21].

**Figure 1:**
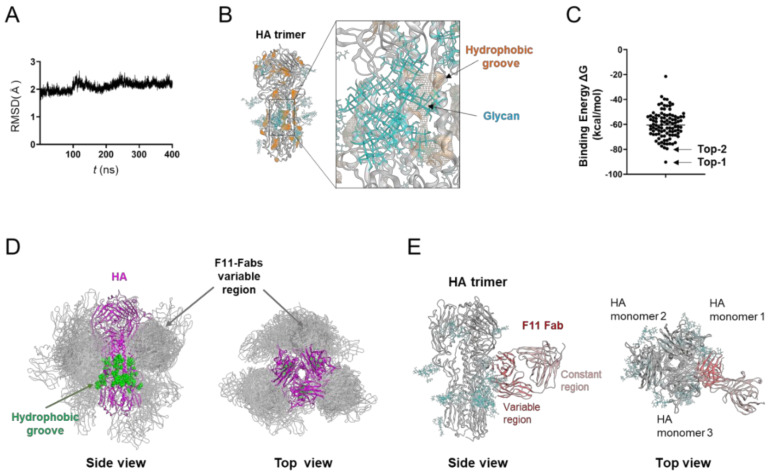
Molecular modeling of the complex of glycosylated HA trimer and F11 Fab fragment. A three-dimensional model of the unliganded, glycosylated HA trimer ectodomain of A/Narita/1/2009 (H1N1)pdm09 virus [19] was constructed by the homology modeling method using the Molecular Operating Environment (MOE) (Chemical Computing Group Inc., Montreal, QC, Canada), glycosylated using tools in GLYCAM-Web [21], and subjected to MD simulation using modules in the Amber 16 program package [23]. The HA trimer model in the equilibrium state under solution conditions was used for the docking simulations of the F11 Fab fragment using the Dock application of MOE [29,30]. (**A**) Root mean square deviation (RMSD) [23] between the structure of the initial HA structure and those at given time points of the MD simulation of glycosylated HA trimers calculated using the cpptraj module in AmberTools 16 as described previously [22]. (**B**) Side view of the HA trimer model at 400 ns of MD simulation. (**C**) Distribution of binding energies among the top 100 binding poses generated using the Dock application of MOE [29,30]. (**D**) Superposition of 100 docking poses. The purple region indicates the HA trimer. Green regions indicate the hydrophobic groove [50] in the HA trimer [53,54]. Grey regions indicate F11 Fab variable regions of the top 100 docking poses. Glycans attached on the HA trimer are not shown here in order to highlight the binding mode of the F11 Fab variable region. (**E**) The second highest ranked binding mode of F11 Fab (top-2 binding mode). Cyan branches indicate the high-mannose oligosaccharide Man_5_GlcNAc_2_.

To obtain trimeric HA ectodomain structures in solution, the initial model was subjected to MD simulation. The structural changes after the start of MD simulation were monitored with root mean square deviation (RMSD) [23] between the initial model structure and the structures at given time points of the MD simulation as described for HA trimers of influenza A virus [18,22] (Figure 1A). The RMSDs sharply increased in the beginning and reached a near plateau after 20 ns of the MD simulations. The results suggest that the structural distortions in the initial HA trimer model were relieved shortly after the onset of MD simulation, and reached a state of thermodynamic equilibrium in solution. The MD simulation also disclosed that glycans at the HA stalk domain had partially hidden the hydrophobic groove [50] located near the binding interface of the reported anti-HA stalk antibodies [9,12,13,14,51,52] (Figure 1B). These results suggest that the HA ectodomain in solution preserves a structure that is partially protected against anti-HA stalk antibodies by the HA stalk glycans.

Using the HA trimer model in the equilibrium state under solution conditions, we conducted in silico docking simulation between HA and the F11 Fab fragment (Figure 1C). The distribution of the free energy of binding (ΔG) showed that various docking poses were physicochemically possible. Among the top 100 docking poses, the vast majority of F11 Fab was bound to a region near the hydrophobic groove [50], in the same manner that anti-HA stalk antibodies were previously shown to bind to the trimeric HA ectodomain [9,12,13,14,51,52] (Figure 1D). We then examined individual docking poses. The highest ranked binding mode (Figure 1C, top-1 binding mode) was unlikely to reflect the natural binding mode of the antibody, since the constant region of Fab was involved in the HA binding and the binding mode could cause a steric clash between the HA head domain and F11 Fc region (Figure S2A). In contrast, only the variable domains of F11 participated in the HA binding and no steric crush was predicted to occur in the binding mode with the second highest binding affinity (Figure 1E and Figure S2B, top-2 binding mode). The top-2 binding mode featured the crosslinking of two HA monomers by F11 Fab variable regions (Figure 1E). The top-2-like binding modes involving HA crosslinking were most frequently seen among the 100 docking poses (Figure S2C). These results suggest that the F11 Fab fragment has physicochemical properties that crosslink two HA monomers by binding to the stalk domain of the glycosylated HA trimer ectodomain.

### 3.2. MD Simulation of Glycosylated HA Trimer Docked to F11 Fab Fragment

**Figure 2:**
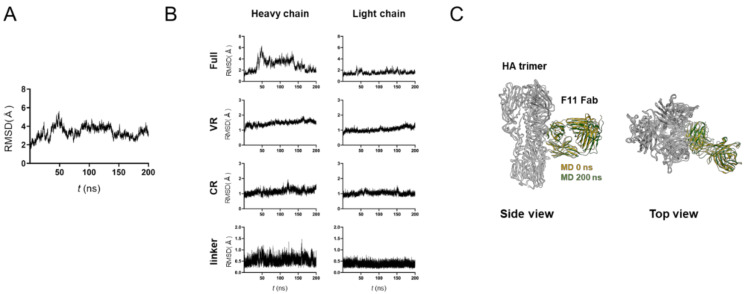
MD simulation of glycosylated HA trimer docked to the F11 Fab fragment. The top-2 complex was subjected to MD simulation at 1 atm and at 310 K in 0.15 M NaCl for 200 ns. (**A**) RMSDs between the structure of the initial HA-F11 Fab complex and those at the given time points of the MD simulation are shown. (**B**) RMSDs of structural units of the F11 Fab fragments during the MD simulations. The entire region (Full), variable region (VR), constant region (CR), and unstructured linker region between VR and CR (linker) are shown for the heavy chain (left panels) and light chain (right panels). (**C**) Superposition of the top-2 complex structures before and after 200 ns of MD simulations.

To assess the stability of the HA-F11 Fab complex, we ran an MD simulation using the top-2 binding mode (Figure 2). The RMSDs sharply increased after the onset of the MD simulations, reaching a near plateau after 100 ns, but with greater levels of fluctuations as compared with those of the unliganded HA trimer (Figure 1A and Figure 2A). This structural fluctuation mainly reflected that of the F11 Fab linker region between the variable region (VR) and constant region (CR), and fluctuations of the VR for HA binding were minimal, if any (Figure 2B). Consequently, no major shifting in the binding site was detected before and after the MD simulation (Figure 2C). These results suggest that the top-2 binding mode is thermodynamically stable under solution conditions.

### 3.3. Characterization of Molecular Interactions between the HA Trimer Ectodomain and F11 Fab Fragment

**Figure 3:**
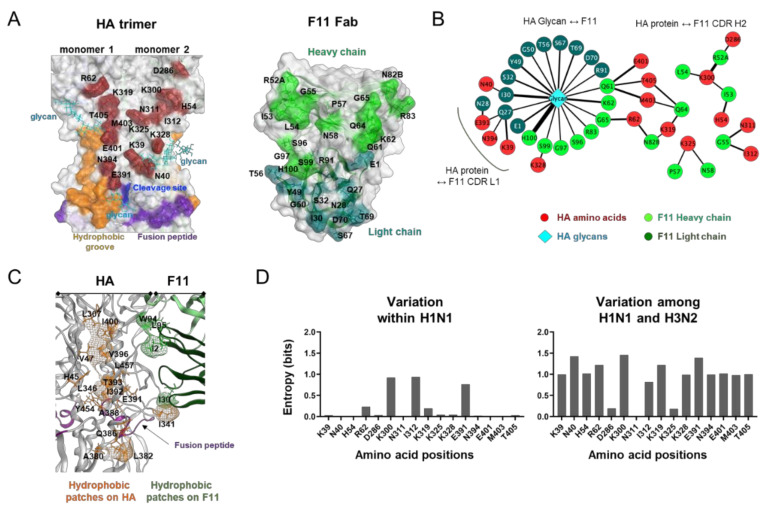
Characterization of molecular interactions between the HA trimer ectodomain and F11 Fab fragment. HA-F11 complex structures collected at every 1 ns during 180 to 200 ns after MD simulations of the top-2 binding mode were used to search for the sites supporting molecular interactions between HA and the F11 antibody using the Contact Analysis application of MOE. (**A**) Visualization of contact sites in the HA trimer (left, wine red residues) and F11 Fab variable region (right, light green and blue green). (**B**) Interaction networks between HA and F11 Fab variable regions. Non-covalent interactions detected during MD simulation were visualized using Cytoscape software version 3.8.2 [34] as described previously [33]. (**C**) Distribution of hydrophobic patches in the binding interface of HA and F11. Hydrophobic patches with a minimum area of 50 Å^2^ for protein-protein interactions were estimated using the Protein Patch Analyzer tool in MOE as described previously [31]. (**D**) Variation of individual amino acid residues forming noncovalent interactions during MD simulation. Amino acid sequences of the HA protein of the influenza A virus H1 (*n* = 36,564) and H3 (*n* = 53,989) were obtained from the GISAID database [42]. Shannon entropy [43] was calculated for individual contact sites detected during MD simulation as described previously [31,41].

The above results suggest that the top-2 complex represents a biologically, physically, and thermodynamically favorable binding mode with the highest binding affinity to the HA ectodomain. Therefore, we further examined details of the molecular interactions using the top-2 structures derived from MD simulation. HA-F11Fab complexes during 180 to 200 ns of MD simulations were used to identify noncovalent interactions between the two molecules (Figure 3). The binding interface of HA during MD simulations consisted of 16 amino acid residues near the hydrophobic groove [50], the proteolytic cleavage site, and the fusion peptide (Figure 3A, left panel). The binding surface of F11 during MD simulations consisted of 20 amino acid residues in the complementarity determining region (CDR) of the heavy and light chains (Figure 3A, right panel). Interactions at individual contact sites could be classified into three groups (Figure 3B): (1) those between three glycans in the HA stalk domain and CDR of F11 heavy/light chains; (2) those between HA and the F11 light chain; and (3) those between HA and the F11 heavy chain. Closely overlapping hydrophobic regions existed around I341 in the HA proteolytic cleavage loop and F11 I30 in the light chain CDR1 region (Figure 3C). These data suggest that the F11 Fab fragment could bind to trimeric HA via long-range interactions near the functional sites of the HA stalk.

HA stalk amino acid residues constituting the contact sites were basically highly conserved within influenza A/H1 viruses except for a few sites (Figure 3D, left panel), whereas they were markedly different between the influenza A/H1 and A/H3 viruses (Figure 3D, right panel). These results suggest that the F11 antibody is primarily effective on A/H1 viruses but not on A/H3 viruses. This prediction is consistent with the previous finding that the F11 IgG antibody effectively blocked infections by A/H1 viruses, but not A/H5 viruses [18].

### 3.4. Effects of F11 Fab Binding on the Structural Fluctuations of the HA Ectodomain

**Figure 4:**
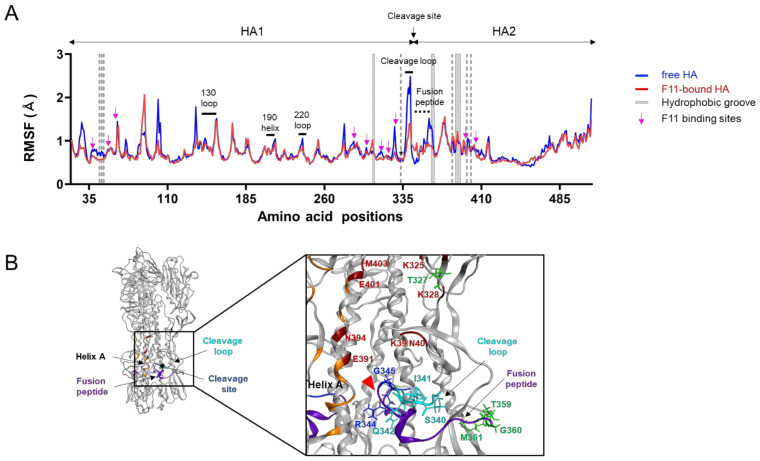
Effects of F11 Fab binding on the structural fluctuations of the HA ectodomain. Free and F11 Fab-bound trimeric HA ectodomains were subjected to MD simulations for 200 ns using the Amber 16 program package [23]. RMSF values of the Cα atoms of individual HA residues were calculated using 10,000 snapshots during 180 to 200 ns of MD simulations as described previously [18,31,32]. (**A**) Distributions of RMSF in HA. Numbers on the horizontal axes indicate positions in the HA of influenza A/Narita/1/2009 (H1N1)pdm09 [19]. Magenta arrows indicate the residues contacting F11 Fab during 180 to 200 ns of MD simulations of the top-2 binding mode. The gray shadows indicate locations of HA residues constituting the hydrophobic groove. Regions involved in sialic acid binding [63,64], such as the 130 loop, 190 helix, and 220 loop, are merged with black bars. (**B**) Structure around the proteolytic cleavage loop. The cleavage loop, fusion peptide [64], and hydrophobic groove [53,54] are shown in cyan, purple, and orange, respectively. A red arrowhead indicates the cleavage site. Residues that noncovalently interacted with the F11 Fab fragment during MD simulation are marked with a wine red color.

Structural fluctuations of biological macromolecules in solution play key roles in molecular interactions [55,56,57,58] and thus in the biological phenotypes of viruses [18,31,32,33]. To gain insights into the structural impact of F11 binding to HA, we compared fluctuations of the HA ectodomain between the free and F11-bound forms using root mean square fluctuation (RMSF), a quantitative indicator of atomic fluctuation [23] (Figure 4). As expected from previous reports [18,31,32], structural fluctuations were often seen in the loops of the ligand-free HA glycoprotein (Figure 4A, free HA). Notably, the proteolytic cleavage loop in the stalk domain, which is the target of cellular proteases [59,60,61,62], was found to fluctuate most heavily in the HA ectodomain under solution conditions. In contrast to the marked impacts on the HA cleavage site, F11 binding to the HA ectodomain did not affect fluctuations in the regions for the HA receptor binding in the head domain [63,64] (Figure 4A, 130 loop, 190 helix, and 220 loop). Interestingly, F11 binding to HA markedly reduced the structural fluctuations around the cleavage loop, as indicated by the marked reduction in the RMSF values (Figure 4A, F11-bound HA). The suppression was detected in the residue at position 327 (T327), residues in the cleavage loop, and residues in the fusion peptide for membrane fusion [50] (Figure 4A). It should be noted that these regions were not direct contact sites of F11, except for the T327 neighboring of an F11 contact residue K328 (Figure 3A and Figure 4B). Instead, the regions that were influenced by the F11 binding were basically assembled to lie within the F11 binding sites (Figure 4B). These results suggest that F11 binding could structurally and physically alter the conformational rearrangement of the HA ectodomain.

### 3.5. In Silico Site-Directed Mutagenesis

**Figure 5:**
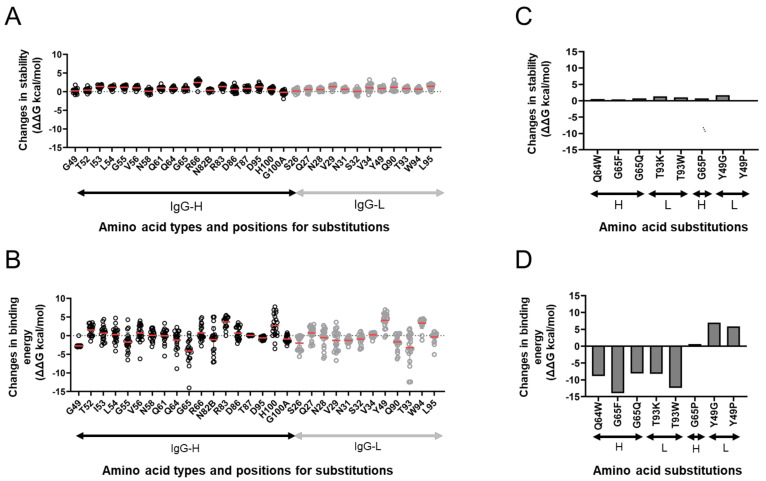
In silico site-directed mutagenesis. F11 Fab residues capable of noncovalently interacting with HA (Figure 3A) and the surrounding residues were individually exchanged for 19 nonself residues, and changes in the stability and binding affinity of F11 Fab were calculated using the F11/HA complex structure obtained at 200 ns of MD simulations as described previously [44,45,46] using the Protein Design application of MOE. (**A**,**C**) Effects on the stability of the F11 Fab fragment. (**B**,**D**) Effects on the binding affinity of the F11 Fab fragment to the HA ectodomain. (**C**,**D**) Results on F11 mutants used for the experimental mutagenesis.

To assess the relative contributions of individual contact residues of F11 to the binding affinity of F11 Fab to the HA stalk domain, we conducted in silico site-directed mutagenesis of the contact sites. The F11 Fab residues that were capable of interacting noncovalently with HA (Figure 3A) and the surrounding residues were individually exchanged with 19 nonself residues, and their effects on F11 stability and activity were analyzed as described previously [44,45,46] (Figure 5). The single substitutions tested had little effect on the stability of the F11 Fab fragment (Figure 5A), as expected from the fact that the substitutions were introduced in the variable region of the antibody. In contrast to the stability, the F11 substitutions had some impact on the calculated binding affinity of Fab to HA, although these effects were minor, mostly consisting of changes in the free energy of binding (ΔΔG) within 5 kcal/mole or less (Figure 5B).

We chose eight single substitutions for further analyses. These F11 substitutions presumably had a different influence on the binding affinity to the HA ectodomain without affecting the F11 stability (Figure 5C,D). Single substitutions at positions 64 and 65 (Q64W, G65F, and G65Q) in the F11 heavy chain, which noncovalently interacted with HA residues near the upper part of the hydrophobic groove during MD simulation (Figure 3A,B), were predicted to augment the binding affinity (Figure 5D). G65P substitution was predicted to cause no major changes in the binding affinity, suggesting that the types of side chain at position 65 are important to maintain the binding affinity. Substitutions at position 93 in the F11 light chain, which exhibited no noncovalent interactions with HA during MD simulation, somehow augmented the binding affinity. In contrast to these findings, single substitutions at position 49 in the F11 light chain, which noncovalently interacted with glycan on asparagine at position 40 (N40) near the cleavage site of HA (Figure 3A,B), were predicted to attenuate the binding affinity (Figure 5D).

### 3.6. Experimental Mutagenesis

**Figure 6:**
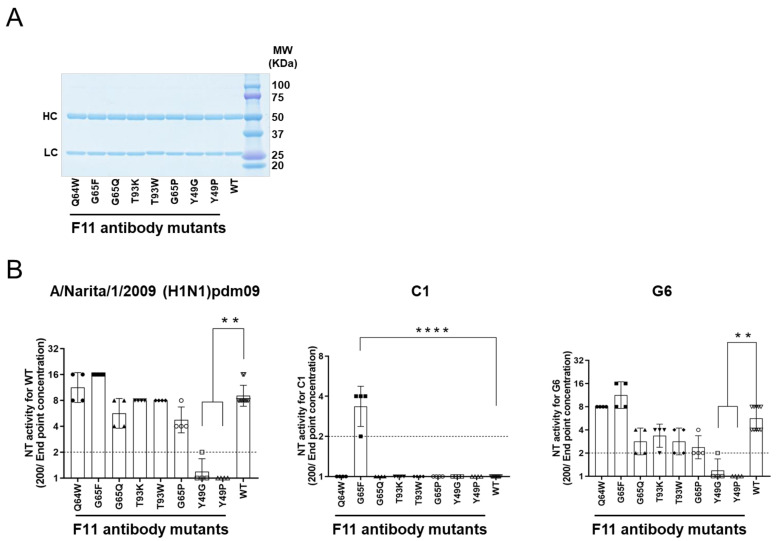
Experimental mutagenesis. Single amino acid substitutions were introduced into the Fab fragment of the F11 IgG1 antibody according to the information in Figure 5C,D. Affinity-purified soluble IgG1 antibodies were used to assess neutralization activity using humanized MDCK cells and 100 TCID_50_ of influenza virus as described previously [48,49]. Neutralization activity was defined as the reciprocal of the lowest concentration (µg/mL) of antibody at which A630 was >X as calculated using the formula X = (A cell − A virus)/2. (**A**) SDS PAGE of the purified F11 IgG fractions used for the neutralization assay. (**B**) Neutralization activity against the F11-sensitive (H1N1)pdm09 virus [18] (left panel), F11-resistant (H1N1)pdm09 variant C1 [18] (middle panel), and F11-weakly resistant (H1N1)pdm09 variant G6 [18] (right panel). NT activity is presented in the scatter plots as the geometric mean, with the geometric standard deviation from four technical replicates (except for F11 WT: *n* = 10). Y-axis values represent neutralizing titers, calculated as 100/minimum concentration (μg/mL) of antibody that neutralized the virus. Dotted lines represent the detection limit (y = 1; 100 μg/mL). ** *p* < 0.01 and **** *p* < 0.0001, comparing F11 mutants with F11 WT (Kruskal–Wallis test, followed by Dunn’s multiple comparison test). For statistical analysis, a provisional minimum NT activity value (y = 0.5; 200 μg/mL) was applied to samples in which NT activity was below the detection limit.

To assess the predictive validity of the in silico mutagenesis, we conducted an experimental mutagenesis study (Figure 6). We assumed that an increase in binding affinity by a given substitution might increase the neutralization activity of F11 against not only the original A/Narita/1/2009 (H1N1)pdm09 virus but also its variants. In this regard, a study on neutralization-resistant viruses might have virological significance. Therefore, we included two A/Narita/1/2009 (H1N1)pdm09 variants here, C1 and G6, that possess single mutations in the HA stalk domain and exhibit different levels of reduced sensitivity to the F11 IgG antibody: C1 has a highly resistant phenotype and G6 has a weakly resistant phenotype [18]. The C1 virus possessed a T333K substitution that has been reported to be involved in resistance to a broad range of anti-HA stalk antibodies in infection of MDCK cells by the A/Netherlands/602/2009 (H1N1)pdm09 virus [10].

Purified IgG1 fractions of F11-derived mutants were prepared as described previously [18] and used for a neutralization assay with humanized MDCK cells [48,49]. We confirmed successful production and purification of F11 IgG1 variant antibodies by SDS-PAGE (Figure 6A), as expected from in silico data on the stability of F11 Fab mutants (Figure 5C). Individual F11 mutations appeared to have distinct effects on the neutralization activity against the A/Narita/1/2009 (H1N1)pdm09 virus (Figure 6B, left panel). G65F in the F11 heavy chain, which was predicted to achieve the greatest increase in the binding affinity to HA (Figure 5D), consistently increased the neutralization activity of F11 by about two-fold, although statistical significance was not obtained (Figure 6B, G65F in the left panel). More remarkably, single substitutions at position 49 in the F11 light chain, which was predicted to moderately reduce the F11 binding affinity to HA (Figure 5D), consistently reduced F11 neutralization activity (Figure 6B, Y49G/P in the left panel). No statistically significant changes were detected with the other F11 mutants.

As expected from the previous study [18], the purified F11 IgG1 fraction had little neutralization activity against the F11-resistant C1 virus (Figure 6B, WT in the middle panel). Most of the F11 single substitutions also had little activity. Notably, however, G65F substitution was found to confer an important new phenotype on F11 IgG1: neutralization activity against the C1 virus (Figure 6B, G65F in the middle panel. The G65F substitution also had positive effects on activity against the G6 virus, as it did against the original virus (Figure 6B, G65F in the left and right panels). Here again, a marked reduction in neutralization activity was detected with F11 possessing Y49G/P substitutions. No statistically significant changes were detected with the other F11 mutants. Thus, the in silico data on the binding affinity, i.e., F11 substitutions exhibiting the greatest positive and negative effects on in silico binding affinity, were consistent with the positive and negative effects on neutralization activity.

## 4. Discussion

In this study, we characterized the molecular interactions between an anti-HA stalk antibody F11 Fab fragment and the trimeric form of the influenza A(H1N1)pdm09 HA glycoprotein. We then used this information to produce F11 variants with new phenotypes by single substitutions in the F11 variable region. Thus far, there has been only a single report on the structure-based creation of new antibodies against influenza viruses [65]. That earlier study presented a successful case of modification of an anti-neuraminidase antibody. To our knowledge, however, the present study is the first report to describe the creation of an anti-HA stalk antibody possessing neutralization activity against a neutralization-resistant virus.

In this study, we have constructed and characterized 100 complex models of an HA ectodomain docked to an F11 Fab fragment using homology modeling, MD simulation, and docking simulation to understand the structural basis of the actions and modifications of this antibody. Our in silico study with the 100 models disclosed that the F11 Fab fragment has physicochemical features that crosslink two HA monomers by binding to a region near the proteolytic cleavage site of the stalk domain (Figure 1). This unique binding mode is represented by the top-2 docking pose with the lowest free energy of binding and stability during MD simulation (Figure 2). Variation in the HA residues contacting the F11 Fab in this binding mode explains the neutralization specificity of the F11 antibody [18] (Figure 3) well. Moreover, the F11 Fab binding to HA was predicted to have little effect on the structural dynamics of the surfaces on the HA head domain for the sialic acid binding (Figure 4), consistent with our previous observation that the F11 antibody did not suppress the hemagglutination inhibition activity of HA [18]. Finally, F11 derivatives exhibiting positive and negative effects on neutralization activity were effectively obtained with the top-2 binding mode (Figure 5 and Figure 6). Collectively, these results strongly suggest that the binding mode proposed in this study represents a physicochemically, thermodynamically, and virologically reasonable mode for the F11 Fab fragment. To date, similar binding modes involving the crosslinking of two HA monomers have often been reported with anti-HA stalk antibodies [9,12,13,14,51,52]. Thus, the crosslinking is likely to represent a favorable binding mode for the anti-HA stalk antibodies in general.

Results of our present in silico studies suggest that there are at least two types of structural impacts for the F11-mediated neutralization of influenza viruses. First, the F11 binding to HA may disturb the cleavage of HA that is a prerequisite for the ordered conformational rearrangement to establish virus infection of the cells [11,60]. Our study suggests that F11 Fab can bind to a region near the proteolytic cleavage site of the stalk domain (Figure 3) and suppress the dynamics of structural units, such as the cleavage loop, proteolytic cleavage site, and fusion peptide (Figure 4). Because the structural dynamics of the molecular interaction surface is critical for the molecular interactions and thus the function of biomolecules [18,31,32,33,55,56,57,58], it is possible that F11 binding not only sterically but also physically inhibits the proteolysis of HA by cellular proteases. In this regard, it is worth noting that proteolysis of HA is suggested to occur with proteases in human respiratory epithelial cells [59,66]. Such proteolysis can occur with HA on the virion by the cell surface human airway trypsin-like protease [59,66] prior to endocytosis. Therefore, F11 binding to HA on the incoming virion outside of the cells may be able to disturb the proteolytic cleavage during virus entry and consequently block initiation of the conformational rearrangement of HA in the cells.

Alternatively, the F11 binding to HA may inhibit pH-dependent conformational changes in the endosomes that are required for viral membrane fusion and genome uncoating in the cells [11,50,61,67]. Our in silico study suggests that the F11 Fab fragment can crosslink two HA monomers by binding to a conserved region near the proteolytic cleavage site of the stalk domain (Figure 3). This binding mode should sterically prevent the conformational changes in endosomes, such as exposure of the fusion peptide, and thus block subsequent processes, such as membrane fusion and genome uncoating, in the case that F11 maintained its binding ability to HA under acidic conditions. Further study is necessary to address each of these possibilities.

Next, we attempted to modify F11 based on the structural information related to the F11 Fab-trimeric HA ectodomain interactions. Structure-guided mutagenesis of the F11 antibody led us to identification of a residue 49 in the F11 light chain as a critical site for the F11-mediated neutralization (Figure 5 and Figure 6). Single amino acid substitutions at this site almost completely abolished the neutralization activity of F11. Reduction in the binding affinity was constantly detected for each of the complexes during the MD simulations, suggesting the affinity reduction could occur in solution (Figure S3). However, it is hard to explain the complete abolishment of F11 neutralization activity by affinity reduction alone because the levels of reduction were rather small, and because considerable affinities remained during the MD simulation (Figure S3). Therefore, it is likely that additional factors in addition to the affinity reduction are involved in the dysfunction of F11 possessing the single substitutions at position 49. In this regard, it should be noted that the F11 residue at position 49 presumably interacts noncovalently with an HA glycan moiety on asparagine at position 40 (N40) near the HA cleavage site by forming a hydrogen bond, and that a single substitution at position 49 of F11 could result in the loss of this interaction (Figure S3). These results suggest that the free glycan moiety on N40 is critical for virus infection of cells. Nonetheless, the function of this glycan in viral protection against the anti-HA stalk antibody [14,68], as well as its function in virus infection and replication have not been reported thus far. Further study on the function of this particular glycan near the HA cleavage site may provide new insights into the mechanisms of influenza virus infection and replication. It is important to study such resistance mutations and develop methods to overcome the resistance, because antibodies targeting the stalk domain can be a powerful tool to combat infections of influenza viruses [6,7,8].

We previously isolated two A(H1N1)pdm09 variants, the C1 and G6 viruses, that exhibited potent and weak resistance to F11, respectively [18]. Interestingly, a recent study has shown that T333K, a single substitution detected in the C1 virus, is involved in resistance to a broad range of anti-HA stalk antibodies [9,10], indicating that the residue is a key player for the anti-HA stalk antibody resistance. Curiously, a virus possessing the T333K substitution still retained an ability to bind to the anti-HA stalk antibodies FI6 [10]. The results indicate that the T333K substitution functions in the resistance by mechanisms that are not involved in the prevention of antibody binding. One such mechanism may be the T333K-induced restoration of dysregulated structural dynamics of HA caused by antibody binding. In this study, we have suggested by MD simulation that F11 antibody binding to the trimeric HA induces dysregulation of structural dynamics in the fusion loop and its flanking regions (Figure 4). This structural defect may be restored by the T333K substitution, so that proteolytic cleavage proceeds to initiate conformational changes for infection. Further studies involving MD simulation will help to address this issue. 

Our structure-guided mutagenesis of the F11 antibody in this study led to identification of an F11 derivative that suppresses infections by the C1 and G6 viruses (Figure 5 and Figure 6). Importantly, G65F single substitution in the F11 heavy chain was sufficient to overcome the resistance (Figure 6B). At present, the molecular mechanism underlying this phenomenon remains unclear. A possible mechanism might be a dysfunction of the HA moieties by G65F substitution. The F11 residue at position 65 in the heavy chain presumably contacts the amino acid and glycan residues near the hydrophobic groove (Figure 3A,B). Thus far, the functions of these HA moieties in virus infection and replication have not been reported. Interestingly, the G65F substitution was suggested to induce drastic changes in the interaction networks around R62, K319, and M403 (Figure S3B), which may suppress the structural dynamics around these residues. Further structure–functional studies on these particular amino acids and glycan residues may provide new insights into the mechanisms of influenza virus infection and replication for antibody modification.

In conclusion, we here proposed a physicochemically, thermodynamically, and virologically reasonable model of the anti-HA stalk antibody F11 and trimeric HA ectodomain. The model involves crosslinking of two HA monomers and has provided new clues for understanding the structural mechanisms of virus neutralization by the F11 anti-HA stalk antibody. Moreover, the model was found to be useful to identify a critical site for the F11-mediated neutralization, as well as to generate anti-HA stalk antibodies that acquired the ability to suppress infections of F11-resistant viruses. Since the anti-HA stalk antibody is a potential tool to combat the influenza virus [6,7,8], it would be important to understand the mechanisms that underlie the neutralization of neutralization-resistant viruses using the anti-HA stalk antibody derivative obtained in this study.

## Data Availability

Please contact the corresponding authors (O.K. and S.S.) for data on the present study.

## Supplementary Materials

The following are available online at https://www.mdpi.com/article/10.3390/v13091733/s1: Figure S1: Amino acid sequences of the F11 Fab fragment and HA protein of the influenza A/Narita/1/2009 (H1N1)pdm09 strain; Figure S2: Binding modes in the docking simulations of the F11 Fab fragment and glycosylated HA trimer of influenza A/Narita/1/2009 (H1N1)pdm09; Figure S3: Effects of the F11 Y49P and G65F substitutions on molecular interactions.

## Abbreviations

CDR	complementarity determining region
CR	constant region
DMEM	Dulbecco’s Modified Eagle’s Medium
EHT	Extended Huckel Theory
HA	Hemagglutinin
hCK	Humanized MDCK
MD	Molecular dynamics
MDCK	Madin-Darby canine kidney
MEM	Minimum essential medium
MOE	Molecular Operating Environment
NT	microneutralization
RMSD	Root mean square deviation
RMSF	Root mean square fluctuation
VR	variable region
