# Supplementary material for: Structure-Guided Creation of an Anti-HA Stalk Antibody F11 Derivative That Neutralizes Both F11-Sensitive and -Resistant Influenza A(H1N1)pdm09 Viruses
Source: Viruses. 2021 Aug 31;13(9):1733. doi: 10.3390/v13091733 (PMC8473006; doi:10.3390/v13091733)
Supplement: Supplementary file 1 [file viruses-13-01733-s001.zip › viruses-1340920-SI.pdf]

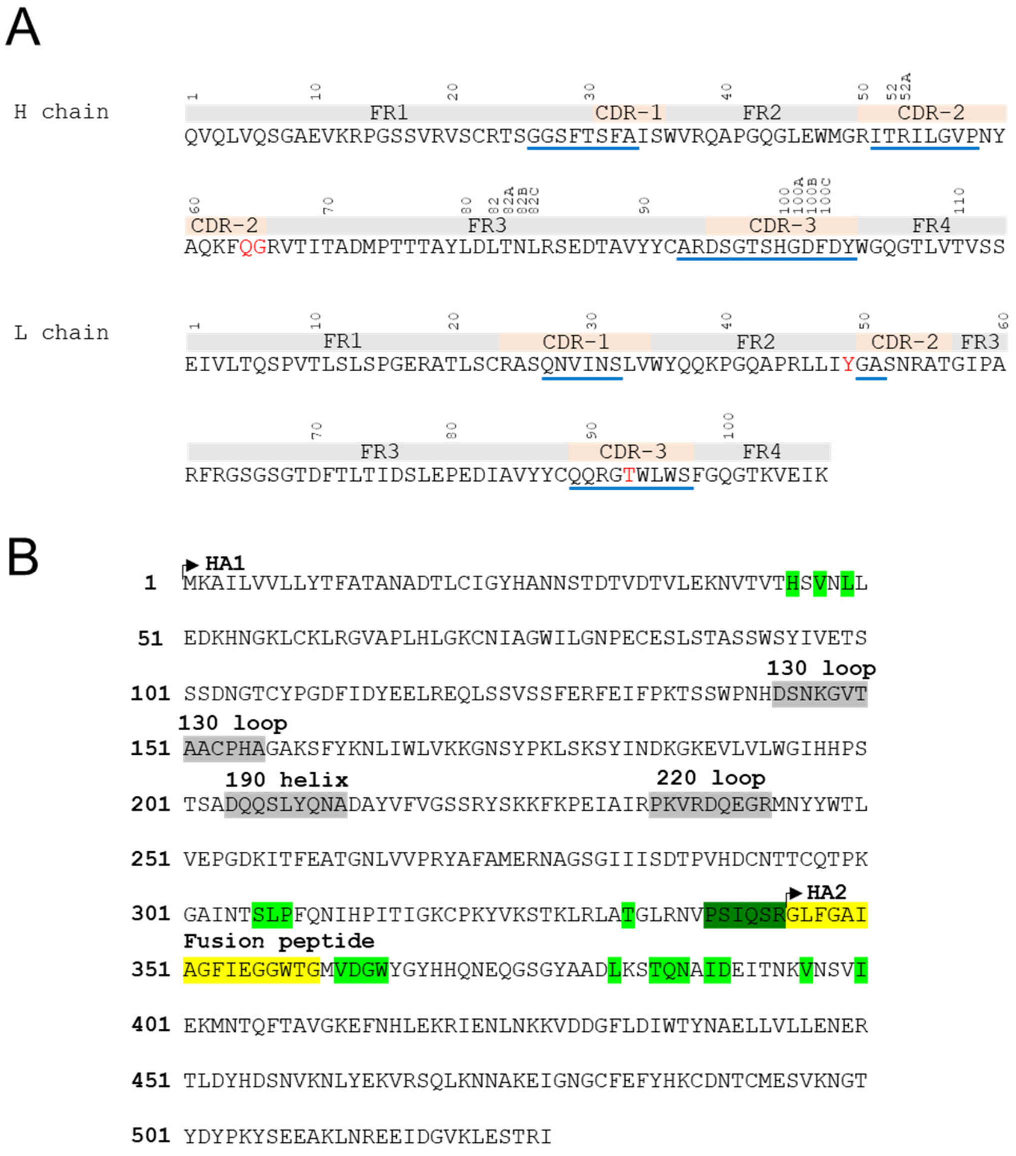

Figure S1. Amino acid sequences of the F11 Fab fragment and HA protein of the influenza A/Narita/1/2009 (H1N1)pdm09 strain. (A) Amino acid positions of the F11 immunoglobulin heavy (H) chain (GenBank accession number: QII15721) and light (L) chain (GenBank accession number: QII15722) of the F11 antibody [1]. Residue positions are according to Kabat numbering [2]. FR: frame region; CDR: complementarity determining region. Blue solid lines indicate the CDR according to IMGT [3]. Residues used for the site-directed mutagenesis in this study are marked in red. (B) Amino acid positions of the A/Narita/1/2009 (H1N1)pdm09 virus HA ectodomain (GenBank accession number: ACR09396)[4]. The hydrophobic groove [5,6], receptor binding sites (130 loop, 190 helix, and 220 loop) [7,8], HA0 cleavage loop and fusion peptide are indicated by green, grey, dark green and yellow, respectively.

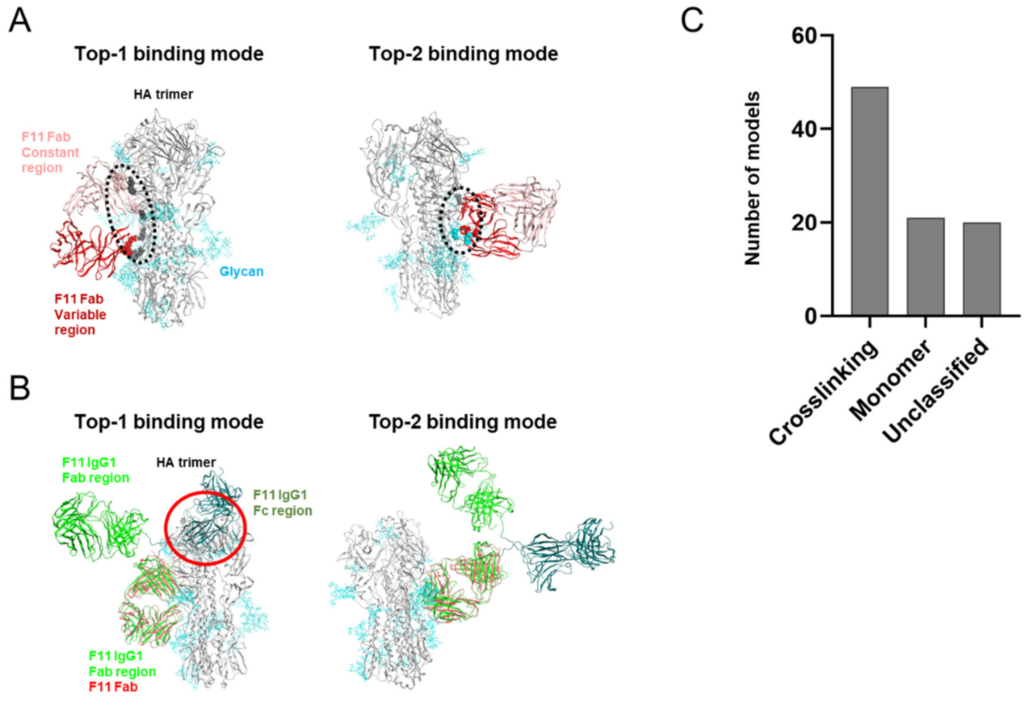

Figure S2. Binding modes in the docking simulations of the F11 Fab fragment and glycosylated HA trimer of influenza A/Narita/1/2009 (H1N1)pdm09. The glycosylated HA trimer model was constructed by homology modeling and subjected to MD simulations using the modules in the Amber 16 program package [9]. The HA structure at 400 ns of MD simulation was used for the docking of the F11 Fab fragment as described in the Materials and Methods. (A) Side views of the top two binding modes. The constant region of Fab is involved in binding in the highest ranked docking pose. Therefore, this docking pose is likely to reflect an artifact, possibly due to the absence of the Fc region in the simulation. Only the variable region of the Fab fragment is involved in the binding in the second highest docking pose (top-2 binding mode). Dotted circles indicate the interaction interface between Fab and HA. (B) Potential steric hindrance in the highest ranked binding mode. The F11 IgG1 full-length model was constructed using the Antibody Modeler application of MOE [10] and superposed with the top two docking poses. A red circle indicates areas of atomic crash between the Fc fragment and HA. (C) Grouping of the F11 binding modes. The top 100 docking poses were grouped according to the binding modes of the Fab fragment. Crosslinking: binding modes that crosslinked two HA monomers; Monomer: binding modes that bound only to HA monomer; Unclassified: binding modes that involved binding of the Fc region or caused steric crashes between F11 antibody and HA.

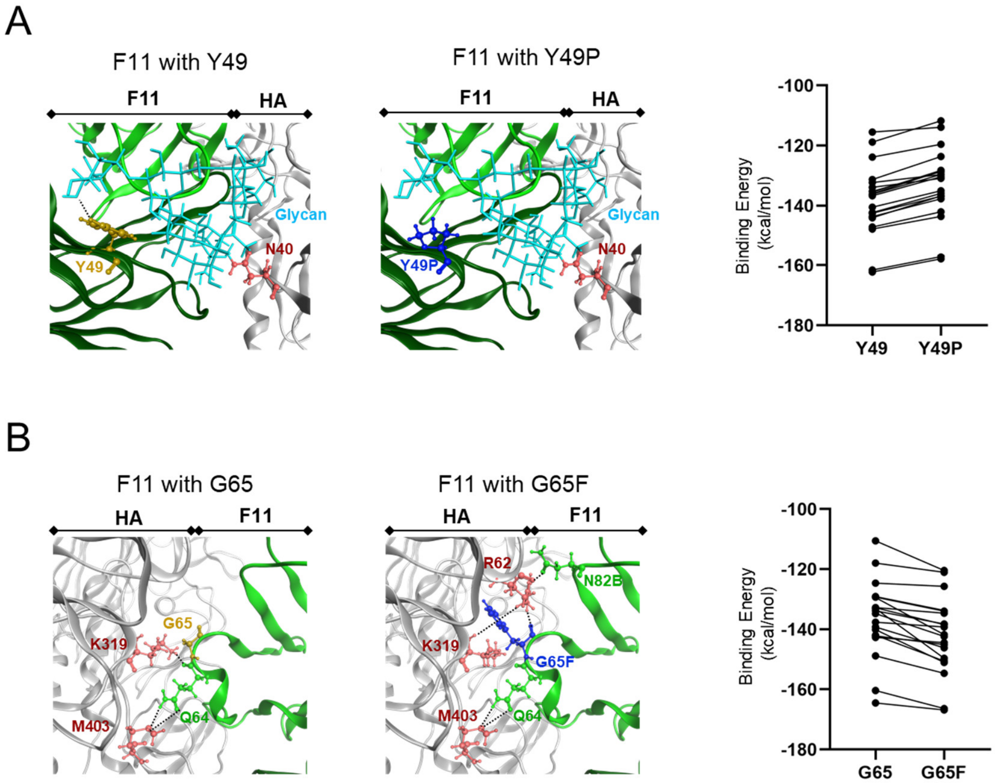

Figure S3. Effects of F11 Y49P and G65F substitutions on molecular interactions. (A, B) Local structures around Y49F (A: left and middle panels) and G65F (B: left and middle panels) substitution sites are shown. Dotted lines indicate hydrogen bonds and arene interaction formed between HA and F11. (A, B: right panel) Distribution of the binding energies of the HA-Fab complexes during MD simulations. Binding energies were calculated with the HA-Fab complexes during 180 to 200 ns of MD simulation (n=20) using the Potential Energy application of MOE.

References

1. Sano, K.; Saito, S.; Suzuki, T.; Kotani, O.; Ainai, A.; van Riet, E.; Tabata, K.; Saito, K.; Takahashi, Y.; Yokoyama, M., et al. An influenza HA stalk reactive polymeric IgA antibody exhibits anti-viral function regulated by binary interaction between HA and the antibody. *PLoS One* **2021**, *16*, e0245244, doi:10.1371/journal.pone.0245244.
2. Wu, T.T.; Kabat, E.A. An analysis of the sequences of the variable regions of Bence Jones proteins and myeloma light chains and their implications for antibody complementarity. *J Exp Med* **1970**, *132*, 211-250, doi:10.1084/jem.132.2.211.
3. Lefranc, M.P.; Giudicelli, V.; Ginestoux, C.; Bodmer, J.; Muller, W.; Bontrop, R.; Lemaitre, M.; Malik, A.; Barbie, V.; Chaume, D. IMGT, the international ImMunoGeneTics database. *Nucleic Acids Res* **1999**, *27*, 209-212, doi:10.1093/nar/27.1.209.

4. Ainai, A.; Hasegawa, H.; Obuchi, M.; Odagiri, T.; Ujike, M.; Shirakura, M.; Nobusawa, E.; Tashiro, M.; Asanuma, H. Host adaptation and the alteration of viral properties of the first influenza A/H1N1pdm09 virus isolated in Japan. *PLoS One* 2015, *10*, e0130208, doi:10.1371/journal.pone.0130208.
5. Dreyfus, C.; Ekiert, D.C.; Wilson, I.A. Structure of a classical broadly neutralizing stem antibody in complex with a pandemic H2 influenza virus hemagglutinin. *J Virol* 2013, *87*, 7149-7154, doi:10.1128/JVI.02975-12.
6. Nath Neerukonda, S.; Vassell, R.; Weiss, C.D. Neutralizing antibodies targeting the conserved stem region of influenza hemagglutinin. *Vaccines (Basel)* 2020, *8*, doi:10.3390/vaccines8030382.
7. Sun, X.; Shi, Y.; Lu, X.; He, J.; Gao, F.; Yan, J.; Qi, J.; Gao, G.F. Bat-derived influenza hemagglutinin H17 does not bind canonical avian or human receptors and most likely uses a unique entry mechanism. *Cell Rep* 2013, *3*, 769-778, doi:10.1016/j.celrep.2013.01.025.
8. Sriwilaijaroen, N.; Suzuki, Y. Molecular basis of the structure and function of H1 hemagglutinin of influenza virus. *Proc Jpn Acad Ser B Phys Biol Sci* 2012, *88*, 226-249, doi:10.2183/pjab.88.226.
9. Case, D.A.; Betz, R.M.; Cerutti, D.S.; Cheatham III, T.E.; Darden, T.A.; Duke, R.E.; Giese, T.J.; Gohlke, H.; Goetz, A.W.; Homeyer, N., et al. AMBER 16. *University of California, San Francisco* 2016.
10. Maier, J.K.; Labute, P. Assessment of fully automated antibody homology modeling protocols in molecular operating environment. *Proteins* 2014, *82*, 1599-1610, doi:10.1002/prot.24576.
